# An Effective Way to Optimize the Functionality of Graphene-Based Nanocomposite: Use of the Colloidal Mixture of Graphene and Inorganic Nanosheets

**DOI:** 10.1038/srep11057

**Published:** 2015-06-08

**Authors:** Xiaoyan Jin, Kanyaporn Adpakpang, In Young Kim, Seung Mi Oh, Nam-Suk Lee, Seong-Ju Hwang

**Affiliations:** 1 Department of Chemistry and Nanoscience, College of Natural Sciences, Ewha Womans University, Seoul 120-750, Korea; 2 National Institute for Nanomaterials Technology (NINT), Pohang University of Science and Technology (POSTECH), Pohang 790-784, Korea

## Abstract

The best electrode performance of metal oxide–graphene nanocomposite material for lithium secondary batteries can be achieved by using the colloidal mixture of layered CoO_2_ and graphene nanosheets as a precursor. The intervention of layered CoO_2_ nanosheets in-between graphene nanosheets is fairly effective in optimizing the pore and composite structures of the Co_3_O_4_–graphene nanocomposite and also in enhancing its electrochemical activity via the depression of interaction between graphene nanosheets. The resulting CoO_2_ nanosheet-incorporated nanocomposites show much greater discharge capacity of ~1750 mAhg^−1^ with better cyclability and rate characteristics than does CoO_2_-free Co_3_O_4_–graphene nanocomposite (~1100 mAhg^−1^). The huge discharge capacity of the present nanocomposite is the largest one among the reported data of cobalt oxide–graphene nanocomposite. Such a remarkable enhancement of electrode performance upon the addition of inorganic nanosheet is also observed for Mn_3_O_4_–graphene nanocomposite. The improvement of electrode performance upon the incorporation of inorganic nanosheet is attributable to an improved Li^+^ ion diffusion, an enhanced mixing between metal oxide and graphene, and the prevention of electrode agglomeration. The present experimental findings underscore an efficient and universal role of the colloidal mixture of graphene and redoxable metal oxide nanosheets as a precursor for improving the electrode functionality of graphene-based nanocomposites.

Graphene-based nanocomposite is one of the most currently investigated materials in the fields of chemistry, physics, materials science, and nanotechnology because of its intriguing physicochemical properties and promising functionalities^1^,^2^,^3^,^4^. This family of materials boasts excellent functionalities for many energy-related applications such as secondary batteries, supercapacitors, photocatalysts, photovoltaics, and fuel cells^4^,^5^,^6^,^7^,^8^,^9^,^10^,^11^,^12^. One of the most promising applications of the graphene-based nanocomposites is an electrode for secondary batteries. An increasing demand for the large-scale application of secondary batteries evokes intense research efforts for the exploration of novel graphene-based electrode materials showing excellent rate characteristics and high electrochemical stability^11^. The hybridization of electrode materials with highly conductive graphene nanosheets leads to a significant improvement of electrode performance at high current density via the increase of electrical conductivity^12^. Additionally the porous stacking structure of the graphene-based nanocomposite can relieve the drastic volume change and electrical disconnection of electrode materials upon electrochemical cycling, leading to the improvement of cyclability^12^. Thus, there is a great deal of research activity for the nanocomposite electrode materials composed of reduced graphene oxide (rG-O) nanosheets and electrochemically active metal oxides like Co_3_O_4_, Mn_3_O_4_, and SnO_2_ and elemental metals/semimetals like Si and Ge^13^,^14^,^15^,^16^,^17^,^18^. However, the rG-O nanosheet suffers from a strong tendency to form tightly packed structure due to a strong π–π interaction between sp^2^ carbon arrays. This prevents the intimate nanoscale mixing between electrode crystals and rG-O nanosheets, and the formation of the open stacking structure of nanocomposite, which diminish the beneficial effect of the hybridization with rG-O nanosheets.

Currently intense research interest on graphene nanosheet is extended to 2D nanosheets of inorganic solids such as layered metal oxide, layered metal chalcogenide, and layered metal hydroxide^19^,^20^,^21^. Like the rG-O nanosheets, the subnanometer-thick nanosheets of layered inorganic compounds can be synthesized by the chemical exfoliation of their pristine materials^22^,^23^. The obtained metal oxide nanosheets can form homogeneous colloidal mixture with rG-O nanosheets^24^. Taking into account the stiffness and the absence of π electron clouds of these inorganic nanosheets, the incorporation of metal oxide nanosheets is supposed to be effective in enhancing the porosity and homogeneity of metal oxide–rG-O nanocomposite via the weakening of π–π interactions between rG-O nanosheets. Among diverse metal oxide nanosheets, redoxable transition metal oxide nanosheets such as CoO_2_, [Mn_1/3_Co_1/3_Ni_1/3_]O_2_, and MnO_2_ show sufficiently high electrical conductivity and high electrochemical activity^25^,^26^,^27^. Such redoxable metal oxide nanosheets can be suitable additives for optimizing the composite structure, pore structure, and performance of graphene-based nanocomposite electrode materials. Yet at the time of publication of this study, we are aware of no report about the use of the mixed colloidal suspension of layered metal oxide and graphene nanosheets as a precursor for the optimization of the electrode performance of graphene-based nanocomposites for secondary batteries.

Here we report an effective and universal way to improve the electrode functionality of graphene-based nanocomposites using the colloidal mixture of inorganic and graphene nanosheets. The effects of the intervention of layered CoO_2_ nanosheets on the composite structure, pore structure, and the electrode activity of Co_3_O_4_–graphene nanocomposite are systematically investigated. The present strategy is also extended by the incorporation of layered MnO_2_ nanosheets into the Mn_3_O_4_–graphene nanocomposite.

## Results

**Figure 1:**
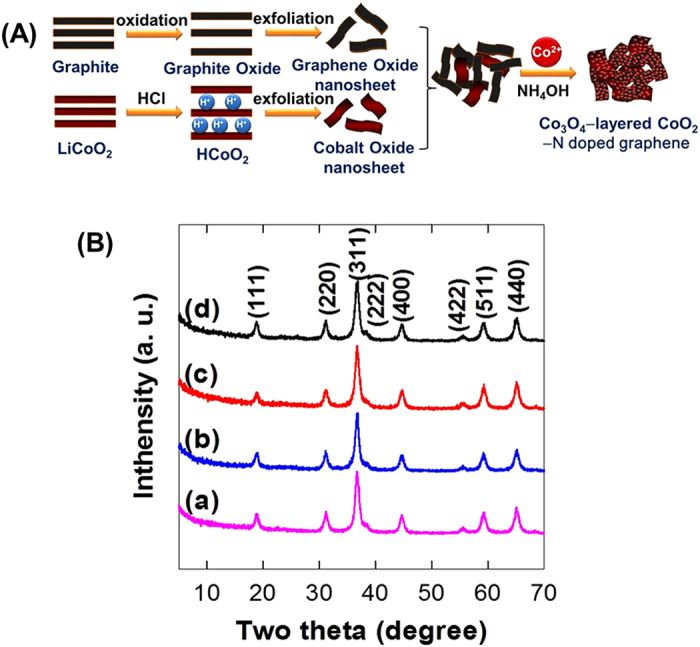
(**A**) Schematic diagram for the synthesis of the **CCG** nanocomposites. (**B**) Powder XRD patterns of (a) **CCG0**, (b) **CCG5**, (c) **CCG10**, and (d) **CCG20**.

The precursors of exfoliated CoO_2_ and G-O nanosheets can form stable mixture colloidal suspensions with variable ratios of CoO_2_/G-O, since they possess very similar surface charge and hydrophilicity each other (Supplementary Information, Fig. S1 and Table S1). The effect of NH_4_OH addition on the colloidal stability of G-O/CoO_2_ mixture as well as on the pure colloidal suspensions of layered CoO_2_ and G-O nanosheets is examined. Upon the addition of NH_4_OH, all the present colloidal suspensions remain unchanged without the formation of aggregated precipitates, clearly demonstrating the excellent colloidal stability of these suspensions (Supplementary Information, Fig. S1A). The size distribution of exfoliated CoO_2_ nanosheet is determined by a standard dynamic light scattering (DLS) analysis (Supplementary Information, Fig. S1B). Most of the exfoliated CoO_2_ nanosheets possess the lateral size of several hundreds of nanometers, which is comparable with the reported lateral dimension of G-O nanosheet^28^. As illustrated in Fig. 1A, the hydrothermal treatment of Co^2+^ ions and NH_4_OH dissolved in the mixture colloidal suspensions of the layered CoO_2_ and G-O nanosheets makes possible the incorporation of layered CoO_2_ nanosheets into the Co_3_O_4_–N-doped rG-O nanocomposite. Since both the exfoliated CoO_2_ and rG-O nanosheets are negatively-charged, the precursor Co^2+^ ions can be easily adsorbed on the surface of both the anionic nanosheets, which is followed by the crystal growth of Co_3_O_4_ phase. The resulting Co_3_O_4_–layered CoO_2_–N-doped rG-O nanocomposites with different CoO_2_/G-O ratios (0, 0.5, 1, and 2wt%) are denoted as **CCG0**, **CCG5**, **CCG10**, and **CCG20**, respectively. The reduction of precursor G-O to N-doped rG-O during the synthesis is confirmed by C 1s and N 1s X-ray photoelectron spectroscopic (XPS) analysis (Supplementary Information, Fig. S2 and Table S2). As presented in the powder X-ray diffraction (XRD) patterns of Fig. 1B, all of the present nanocomposite materials show typical Bragg reflections of spinel-structured Co_3_O_4_ phase, indicating the formation of mixed valent cobalt (II,III) oxide phase during the hydrothermal reaction. On the basis of Scherrer equation, the size of Co_3_O_4_ particle in the present materials is calculated to be 7.7, 8.2, 8.5, and 9.0 nm for **CCG0**, **CCG5**, **CCG10**, and **CCG20**, respectively, highlighting a slight increase of particle size upon the incorporation of layered CoO_2_ nanosheets. The observed minute variation of the particle size of Co_3_O_4_ upon the incorporation of CoO_2_ nanosheets underscores the limited influence of layered CoO_2_ nanosheets on the crystal growth of Co_3_O_4_ nanoparticles.

**Figure 2:**
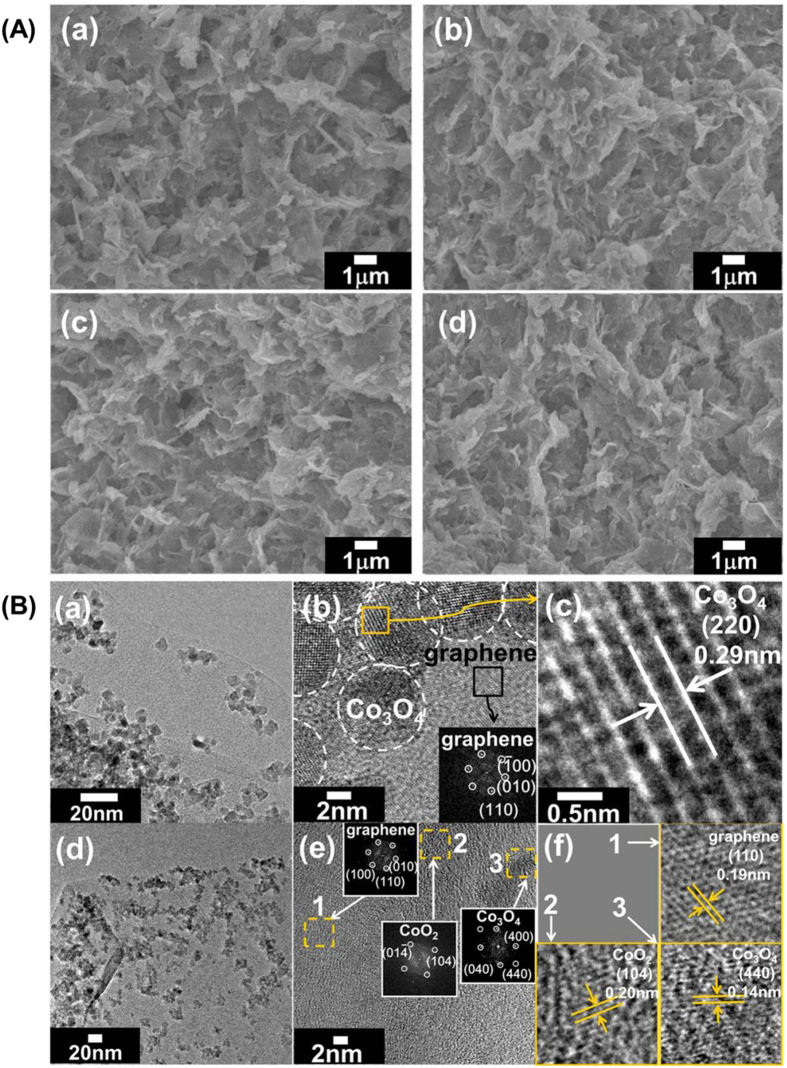
(**A**) FE-SEM images of (a) **CCG0**, (b) **CCG5**, (c) **CCG10**, and (d) **CCG20**. (**B**) TEM/HR-TEM/FFT data of (a,b,c) **CCG0** and (d,e,f) **CCG10** nanocomposites.

As illustrated in the field emission-scanning electron microscopy (FE-SEM) images of Fig. 2A, all the present **CCG** nanocomposites commonly exhibit porous morphology formed by the house-of-cards-type stacking of nanosheet crystallites, indicating the formation of many mesopores. Such a mesoporous stacking structure is commonly observed for the self-assembled nanocomposite materials synthesized by the restacking of 2D nanosheets with 0D nanoparticles^29^. The nanoscale hybridization of cobalt oxide and graphene nanosheets is confirmed by energy dispersive X-ray spectroscopy (EDS)–elemental mapping analysis (Supplementary Information, Fig. S3), showing the uniform distribution of cobalt, oxygen, and carbon in entire parts of the nanocomposite materials.

The composite formation of the rG-O and CoO_2_ nanosheets with Co_3_O_4_ nanocrystals is obviously evidenced by high resolution-transmission electron microscopy (HR-TEM), see Fig. 2B. The fast Fourier transform (FFT) image of the CoO_2_-free **CCG0** material in Fig. 2B–(b) clearly demonstrates the diffraction spots of graphene component. From the enlarged HR-TEM image of the CoO_2_-free **CCG0** material in Fig. 2B–(c), the distance between two consecutive fringes is determined to be ~0.29 nm, which is in good agreement with the interplanar distance of {220} planes of Co_3_O_4_ phase, indicating the immobilization of the spherical Co_3_O_4_ particles on the surface of the nanosheets. For the case of CoO_2_–incorporated **CCG10** nanocomposite, the FFT images of Fig. 2B–(e) provide clear evidence for the co-existence of layered CoO_2_ nanosheets with Co_3_O_4_ and graphene. This is further confirmed by the observation of clear lattice fringes corresponding to the {110} plane of graphene, the {104} plane of layered CoO_2_, and the {440} plane of Co_3_O_4_, as illustrated in Fig. 2B–(f). The present HR-TEM/FFT results obviously demonstrate the homogeneous hybridization of layered CoO_2_, graphene, and Co_3_O_4_ in the present **CCG10** nanocomposite. As a reference, the rG-O-free Co_3_O_4_–layered CoO_2_ nanocomposite is also synthesized by the same synthetic method as that for the **CCG** nanocomposites except for G-O and ammonia. Like the **CCG** nanocomposites, the obtained Co_3_O_4_–layered CoO_2_ nanocomposite displays the formation of spinel-structured Co_3_O_4_ nanoparticles anchored on the surface of layered CoO_2_ nanosheets (Supplementary Information, Fig. S4). This result confirms that, like the N-doped rG-O nanosheet, the layered CoO_2_ nanosheets can play a role of support for the anchoring of Co_3_O_4_ nanoparticles. During the anchored growth of Co_3_O_4_ phase, the layered CoO_2_ nanosheets remains intact without any notable damage. This result provides clear evidence for the high stability of layered CoO_2_ nanosheets against the hydrothermal synthesis.

**Figure 3:**
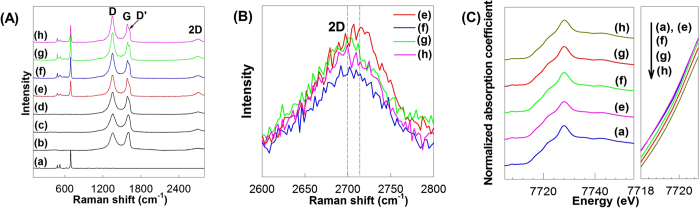
(**A**) Micro-Raman spectra, (**B**) their expanded views of high wavenumber region, and (**C**) Co K-edge XANES spectra and their expanded views near edge jump for (a) Co_3_O_4_, (b) G-O, (c) rG-O, (d) N-doped rG-O, (e) **CCG0**, (f) **CCG5**, (g) **CCG10**, and (h) **CCG20**.

The chemical bonding nature of rG-O and cobalt oxide components in the present nanocomposites is examined with micro-Raman spectroscopy. As illustrated in Fig. 3A, all the present nanocomposites show two intense Raman features D and G in high wavenumber region of >1000 cm^−1^, characteristic of graphene species, confirming the incorporation of graphene nanosheets in these materials^30^. In contrast to N-undoped G-O and rG-O nanosheets, all the **CCG** nanocomposites as well as N-doped graphene demonstrate a distinct shoulder peak D', indicating N-doping for graphene component^30^. This peak D' originates from a significant perturbation of the carbon sp^2^ network of graphene upon the incorporation of nitrogen element. The peak 2D reflecting the degree of the structural disorder and stacking of graphene is observed at ~2700 cm^−1^ for all the present materials. As the content of layered CoO_2_ nanosheets increases, this 2D peak shows a slight red-shift with the increase of spectral weight, clearly demonstrating the decreased numbers of stacked graphene layers upon the intervention of layered CoO_2_ nanosheets in-between the graphene nanosheets^31^ (Fig. 3B). This spectral variation provides strong evidence for the weakening of π–π interaction between the graphene nanosheets upon the incorporation of layered CoO_2_ nanosheets, leading to the prevention of the irreversible restacking or agglomeration of graphene nanosheets during electrochemical Li^+^ insertion/extraction. Such a depression of the restacking or aggregation of graphene nanosheets would be beneficial in enhancing the pore structure of the present **CCG** nanocomposites. In the low wavenumber region, typical Raman features of spinel Co_3_O_4_ phase are discernible commonly for all the **CCG** nanocomposites, confirming the formation of Co_3_O_4_ crystals in these materials. The incorporation of layered CoO_2_ nanosheets as well as the formation of Co_3_O_4_ particles in the present **CCG** nanocomposites is cross-confirmed by Co K-edge X-ray absorption near-edge structure (XANES) spectroscopy, see Fig. 3C. As can be seen clearly from the expanded views of edge jump region, a slight but distinct blue shift of edge position is clearly observed after the incorporation of layered CoO_2_ nanosheets, highlighting an increase of average Co oxidation state caused by the increase of CoO_2_ content. This result confirms the HR-TEM results showing the presence of CoO_2_ nanosheets in the present **CCG** nanocomposites. The successful incorporation of tetravalent CoO_2_ nanosheets in the present materials is further evidenced by the Co 2p XPS result (Supplementary Information, Fig. S5), in which the tetravalent Co^4+^ ions are identified and the concentration of Co^4+^ ion increases with increasing the concentration of CoO_2_ nanosheet incorporated. Both the XANES and XPS results provide strong support for the HR-TEM results showing the presence of CoO_2_ nanosheets in the **CCG** nanocomposites (Fig. 2B).

**Figure 4:**
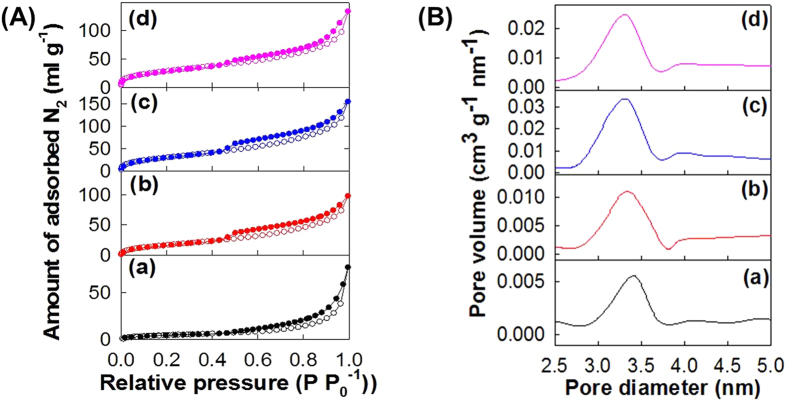
(**A**) N_2_ adsorption–desorption isotherms and (**B**) pore size distribution curves calculated on the basis of the BJH equation for the nanocomposites of (a) **CCG0**, (b) **CCG5**, (c) **CCG10**, and (d) **CCG20**.

As plotted in Fig. 4A, N_2_ adsorption−desorption isotherm measurements clearly demonstrate the porous nature of the present **CCG** nanocomposites. All of the present materials display a significant N_2_ adsorption at low pressure region of pp_o_^−1^ < 0.4, reflecting the existence of micropores in these materials. A distinct hysteresis commonly occurs at high pressure region of pp_o_^−1^ > 0.45 for all the present **CCG** nanocomposites. The observed isotherm behavior corresponds to Brunauer–Deming–Deming–Teller (BDDT) type-IV shape and IUPAC H2-type hysteresis loop, suggesting the presence of open slit-shaped capillaries with very wide bodies and narrow short necks. The incorporation of layered CoO_2_ nanosheets enhances the adsorption of N_2_ molecule in the low pressure region and also the total amount of N_2_ molecules adsorbed, underscoring the remarkable increase of micropore volume and surface area. According to the calculation of surface area using the Brunauer–Emmett–Teller (BET) equation, the surface area of the present nanocomposite is estimated to be 32 m^2^g^−1^ for **CCG0**, 64 m^2^g^−1^ for **CCG5**, 97 m^2^g^−1^ for **CCG10**, and 82 m^2^g^−1^ for **CCG20**, respectively. This result demonstrates that the surface areas of the present **CCG** nanocomposites become greater with increasing the content of CoO_2_ nanosheets upto the composition of **CCG10**. However, the further addition of CoO_2_ nanosheets leads to the depression of surface area. The observed lowering of the surface area of the **CCG20** nanocomposite is attributable to too high content of CoO_2_ nanosheet, which is much heavier than the graphene. That is, the increase of the sample mass caused by the addition of heavy CoO_2_ nanosheets outweighs the accompanying optimization of the pore structure of the nanocomposite. This result clearly demonstrates that, even at a small concentration of CoO_2_ nanosheets, the incorporation of layered CoO_2_ nanosheets is fairly useful in expanding the surface area of Co_3_O_4_–graphene nanocomposite. As evidenced by the micro-Raman spectroscopy (Fig. 3B), the incorporation of CoO_2_ nanosheets is effective in depressing the π–π interaction between rG-O nanosheets and also in preventing the formation of tightly packing structure of graphene. Taking into account the fact that the severe self-restacking of graphene nanosheets leads to the remarkable decrease of surface area, the observed increase of the surface area of **CCG** nanocomposites upon the incorporation of CoO_2_ nanosheets can be attributed to the depressed interaction between the graphene nanosheets. In fact, such a prominent increase of the surface area of restacked graphene nanosheets upon the incorporation of inorganic nanosheet is also observed for other cases like Pt–layered titanate–graphene nanocomposite, clarifying the effectiveness of the nanosheet addition in enhancing the porosity of graphene-based nanocomposite^9^. The calculation of pore size based on Barrett–Joyner–Halenda (BJH) method (Fig. 4B) clearly demonstrates that all the present materials possess uniform-sized mesopores with an average diameter of ~3.3–3.4 nm, which are formed by the house-of-cards-type stacking structure of nanosheet crystallites. The BJH analysis reveals the increase of pore volume upon the incorporation of layered CoO_2_ nanosheets, highlighting the positive effect of inorganic nanosheet in enhancing the porosity of graphene-based nanocomposites. The present results of N_2_ adsorption–desorption isotherm analysis clearly demonstrate that the incorporation of CoO_2_ nanosheets is quite powerful in increasing the surface area and pore volume of the restacked graphene nanosheets via the depression of π–π interaction between the graphene nanosheets.

**Figure 5:**
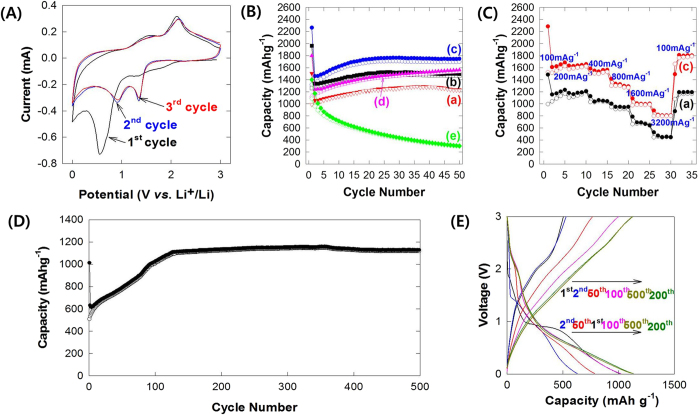
(**A**) CV curve of **CCG10**, (**B**) discharge–charge capacity plots at current density of 200  mA g^−1^ and (**C**) rate-dependent capacity plots of (a) **CCG0**, (b) **CCG5**, (c) **CCG10**, (d) **CCG20**, and (e) layered CoO_2_ nanosheets. (**D**) discharge–charge capacity plots of **CCG10** electrode at current density of 1000 mA g^−1^. (E) Potential profile at current density of 1000 mAg^−1^ for the nanocomposite of **CCG10**.

The present **CCG** nanocomposites are applied as anode materials for lithium ion batteries. Fig. 5A shows the representative cyclic voltammogram (CV) curves of the **CCG10** electrode, which are collected at a scan rate of 0.5 mV s^−1^ in the voltage range of 0.01–3 V vs. Li/Li^+^. In the first cycle, an irreversible reduction peak appears at ~0.7 V, which originates from the degradation of electrolyte caused by the formation of polymer/gel-like film around the electrode particles^32^. In the second cycle, there are two cathodic peaks at 1.41 and 0.9 V, which are ascribed to the reduction of Co_3_O_4_ to Co caused by the lithiation of Co_3_O_4_. The lithiation voltage of the second cycle is shifted to higher value than that of the first cycle, indicating the improved kinetics of the **CCG10** nanocomposite^12^. Meanwhile, two anodic peaks at 1.46 and 2.15 V are attributable to the oxidation of Co element to Co_3_O_4_, which corresponds to the delithiation process. These redox peaks can be regarded as evidence for the electrochemical reaction of Co_3_O_4_ and Li^12^. Although very small quantity of layered CoO_2_ nanosheet makes it difficult to directly detect a redox peak corresponding to reaction between layered CoO_2_ and Li in the present CV data, the lithium insertion/extraction reactions in the present nanocomposites can be described by the following equations.


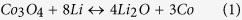



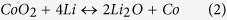


Of prime importance is that there is no significant difference in the CV data of the **CCG10** nanocomposite for the 2nd and 3rd cycles, highlighting the good reversibility of this material.

Fig. 5B shows the galvanostatic discharge–charge curves at a current density of 200 mA g^−1^ in the range of 0.01–3 V vs. Li/Li^+^. All the present nanocomposites exhibit promising electrode performance with the huge initial discharge capacity of 1505, 1963, 2262, and 1797 mAh g^−1^ for **CCG0**, **CCG5**, **CCG10**, and **CCG20**, respectively. Although notable capacity fading occurs at the second cycle due to the formation of solid–electrolyte–interphase (SEI) layer^32^, the discharge capacity of **CCG** nanocomposites becomes greater with proceeding the cycle. Such an increase of discharge capacity is frequently observed for porous nanostructured materials, which is related to the formation of stable diffusion paths of Li^+^ ions during the repeated electrochemical cycling^12^,^33^,^34^,^35^. Among the present nanocomposites, the **CCG10** nanocomposite with the largest surface area exhibits the most prominent enhancement of discharge capacity during the cycle. After the 20th cycle, the discharge capacities of **CCG** nanocomposites are stabilized to ~1230 mAh g^−1^ for **CCG0**, ~1500 mAh g^−1^ for **CCG5**, ~1750 mAh g^−1^ for **CCG10**, and ~1530 mAh g^−1^ for **CCG20**, highlighting the promising electrode performance of the present nanocomposites with huge discharge capacity and good cyclability. To the best of our knowledge, the observed discharge capacity of the **CCG10** nanocomposite is the largest reversible capacity of Co_3_O_4_-based materials ever-reported (Supplementary Information, Table S3). Since all of the components in the present nanocomposite including N-doped rG-O nanosheet are electrochemically active^36^, the observed huge capacity of the present material is a result of the synergistic combination of these electrochemically active materials. On the basis of the theoretical capacities of the component materials (i.e. 890 mAhg^−1^ for Co_3_O_4_, 1216 mAhg^−1^ for N-doped rG-O, and 1178 mAhg^−1^ for CoO_2_)^12^,^35^, the theoretical capacity of the present **CCG10** nanocomposite is estimated to be ~960 mAhg^−1^, which is much smaller that the observed reversible capacity of ~1750 mAhg^−1^. In fact, there are several reports about the larger capacity of graphene-based nanocomposite than the theoretical one^33^,^34^,^37^. On the basis of these researches, several factors are supposed to be responsible for the unusually large reversible capacity of the present **CCG** nanocomposite; (1) the expansion of surface area with the increase of pore volume upon the composite formation results in the additional storage of Li^+^ ions in the interfacial site of nanocomposite^38^. (2) The N-doping for the rG-O component makes another contribution to the large discharge capacity of the nanocomposite, since the N-doping can improve the diffusivity of Li ions in the electrode and can create defects in the graphene lattice providing more active sites for Li insertion and increasing Li adsorption energies at the vacancy sites^36^. (3) During the cycling process, the crystalline Co_3_O_4_ nanoparticles are changed to amorphous ones, leading to the formation of more accessible active sites for Li-ion insertion^34^. (4) The graphene nanosheets suffers from a strong tendency to form tightly packed structure due to the strong π–π interaction between sp^2^ carbon arrays. The incorporation of CoO_2_ nanosheets induces the formation of more open stacking porous structure providing more active sites for Li^+^ ions insertion^9^.

All the present **CCG** nanocomposites display high coulombic efficiency of >98%, reflecting the highly stable and reversible insertion/extraction of lithium ions. As shown in the potential profiles of the **CCG** nanocomposites (Supplementary Information, Fig. S6), all the present materials show nearly identical potential profiles, indicating the retention of the original electrochemical properties of Co_3_O_4_–graphene nanocomposite upon the incorporation of CoO_2_ nanosheets. Based on the present results of electrochemical measurements, it can be concluded that the incorporation of layered CoO_2_ nanosheets leads to the remarkable improvement of the electrode performance of Co_3_O_4_–rG-O (i.e. **CCG0**) nanocomposite.

As presented in Fig. 5C, the remarkable improvement of electrode performance upon the incorporation of layered CoO_2_ nanosheet is more distinct for higher current density condition. The CoO_2_-incorporated **CCG10** nanocomposite exhibits larger reversible capacities for all the current densities applied than does the CoO_2_-free **CCG0** material; the **CCG10** nanocomposite shows the discharge capacities of 1684, 1656, 1562, 1288, 1002 and 817 mAh g^−1^ at the current density of 100, 200, 400, 800, 1600 and 3200 mA g^−1^, respectively. However, the CoO_2_-free **CCG0** electrode delivers much smaller discharge capacities of 1232, 1207, 1044, 949, 642 and 428 mAh g^−1^ at the same current densities, respectively. While the discharge capacity of the **CCG10** nanocomposite at 100 mA g^−1^ is larger by 136% than that of the **CCG0** material, the **CCG10** material shows even twice larger discharge capacity compared with the **CCG0** one at a higher current density of 3200 mA g^−1^. This finding provides clear evidence for the improvement of rate performance upon the incorporation of CoO_2_ nanosheets, highlighting the improvement of charge transport property.

To examine the long-term stability of the CoO_2_-incorporated nanocomposite, the extended electrochemical cycling test is carried out for the **CCG10** nanocomposite with high current density of 1000 mA g^−1^ and in the voltage window of 0.01–3 V vs. Li/Li^+^. As plotted in Figs. 5D, E, this material shows an outstanding cyclability and rate capability with the maintenance of large specific capacity of ~1150 mAh g^−1^ upto the 500th cycle. For the entire cycle, the coulombic efficiency of the **CCG10** nanocomposite is well-maintained to ~99%, verifying the high electrochemical stability of this material. The observed beneficial effect of the incorporation of CoO_2_ nanosheets on the discharge capacity of the nanocomposite is attributable to the expansion of surface area and the change of pore structure, resulting in the additional storage of Li^+^ ions in interfacial site of the CoO_2_ nanosheet-scaffolded nanocomposite. The incorporation of layered CoO_2_ nanosheets also induces an enhanced nanoscale mixing between Co_3_O_4_ particles and rG-O nanosheets, which is responsible for the excellent cyclability and rate characteristics of the present CoO_2_-incorporated **CCG** nanocomposites.

**Figure 6:**
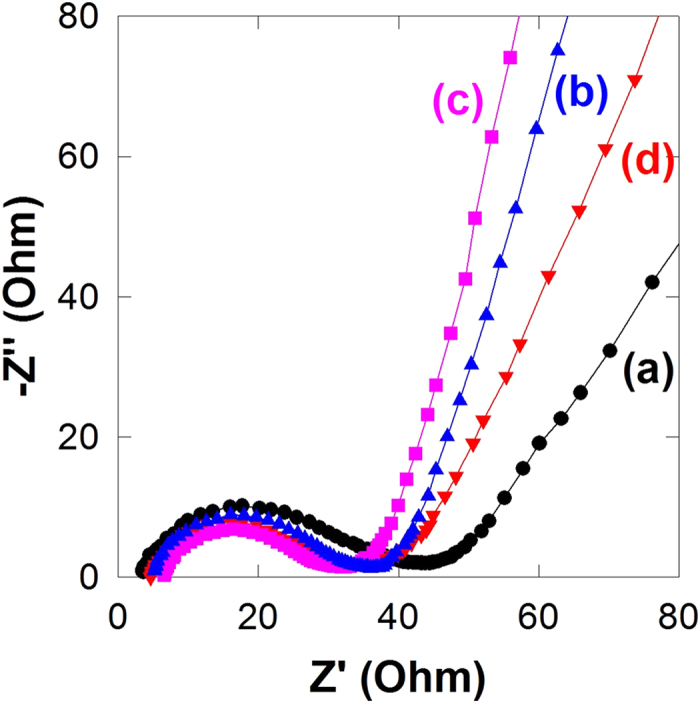


To better understand the origin of the beneficial effect of CoO_2_ addition, the transport property of the present nanocomposites is investigated with electrochemical impedance spectroscopy (EIS). As plotted in Fig. 6, all the present nanocomposites demonstrate partially overlapping semicircles reflecting the charge transfer resistance (R_ct_) at high-to-medium frequencies and a line corresponding to Warburg impedance at low frequencies. The incorporation of layered CoO_2_ nanosheets gives rise to a significant reduction in the diameter of semicircle, indicating the decrease of R_ct_. Among the present nanocomposites, the **CCG10** material displays the smallest diameter of the semicircle, indicating its most efficient transport property. A further increase of CoO_2_ content to the **CCG20** nanocomposite degrades the electron transport property, which is attributable to the decrease of highly conductive graphene content. The relative order of R_ct_ is in good agreement with the relative electrode performances of the present nanocomposites, underscoring the main role of the improvement of transport property in enhancing the electrode performance upon the incorporation of CoO_2_ nanosheets. Such a variation of transport properties is further confirmed by the change of the line slope in the low frequency region. The slope of this line for the present nanocomposites becomes steeper in the order of **CCG0** < **CCG20** < **CCG5** < **CCG10**, reflecting the improvement of transport property caused by the promoted nanoscale mixing of Co_3_O_4_ nanoparticles and graphene nanosheets upon the incorporation of CoO_2_ nanosheets.

**Figure 7:**
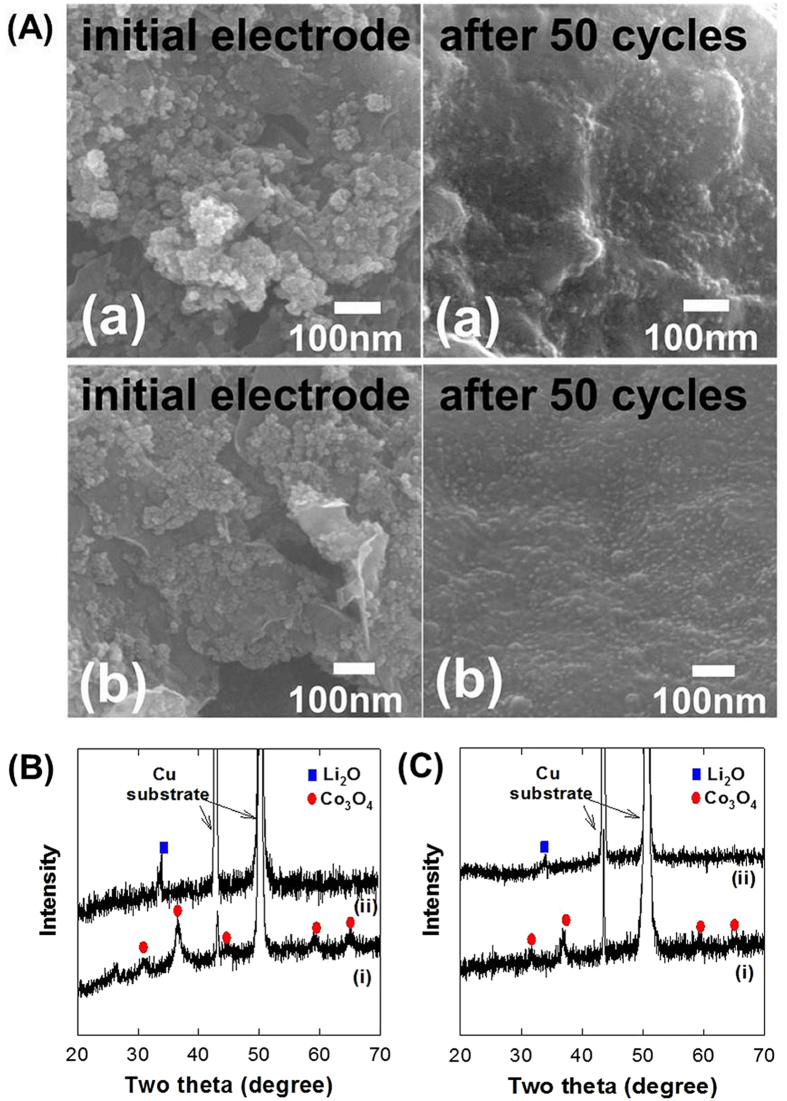
(**A**) Discharge–charge FE-SEM images of (a) **CCG0** and (b) **CCG10**. Powder XRD patterns of (i) as-prepared and (ii) electrochemically-cycled nanocomposites (after the 50^th^ cycle) of (**B**) **CCG0**, and (**C**) **CCG10**.

The effects of electrochemical cycling on the crystal structure and morphology of the present nanocomposites are examined with powder XRD and FE-SEM analyses. As demonstrated in Fig. 7A, the extended electrochemical cycling induces a decrease of the particle size of the **CCG10** nanocomposite whereas the CoO_2_-free **CCG0** nanocomposite shows a significant aggregation of electrode particles. Such an aggregation of electrode particles is negligible for the CoO_2_-incorporated **CCG10** nanocomposite, confirming the beneficial role of CoO_2_ nanosheets in the maintenance of the open structure of nanocomposite. Since Co_3_O_4_ experiences severe volume change during lithiation–delithiation process, the depression of particle agglomeration is surely advantageous in enhancing the electrode performance of the nanocomposite. In addition, the electrochemical cycling induces an amorphization of both the **CCG0** and **CCG10** nanocomposites, see Figs. 7B,C. Such a formation of disordered structure during the electrochemical cycling causes the significant enhancement of Li^+^ diffusion via the provision of more diffusion paths^39^. The incorporation of layered CoO_2_ nanosheets does not induce any significant change in the XRD data of the cycled derivative, indicating negligible effect on the structural stability of the nanocomposite. The present finding strongly suggests that the beneficial role of CoO_2_ addition mainly originates from the improvement of the morphological stability of composite structure rather than the change of crystal structure. Even though the incorporated CoO_2_ nanosheets is transformed into cobalt oxide particles during the electrochemical cycling, an intimate mixing between cobalt oxide and rG-O in the CoO_2_-incorporated **CCG10** nanocomposite provides improved diffusion paths for Li^+^ ions as well as a strong electronic coupling between cobalt oxide and graphene nanosheeets.

Such an advantageous effect of the incorporation of metal oxide nanosheet is further evidenced from MnO_2_ nanosheet-incorporated Mn_3_O_4_–N-doped rG-O nanocomposite. The obtained Mn_3_O_4_–layered MnO_2_–N-doped rG-O displays the typical XRD patterns of Mn_3_O_4_ phase and porous morphology formed by the house-of-cards-type stacking of sheet-like crystallites, as observed for the present **CCG** nanocomposites (Supplementary Information, Fig. S7). This material shows the discharge capacity of 1383 and 900 mAh g^−1^ for the 1st and 2nd cycles, respectively. The discharge capacity of this material becomes increasing with proceeding the cycling, leading to the huge discharge capacity of 1250 mAh g^−1^ at the 50th cycle. Even at high current density of 2000 mA g^−1^, the material can still deliver a high capacity of ~700 mAh g^−1^ (Supplementary Information, Fig. S7). The electrode performance of the present nanocomposite is the best one among the reported data of Mn_3_O_4_–graphene nanocomposites (Supplementary Information, Table S4), highlighting the excellent electrode activity of the present material. It is worthwhile to mention that, in comparison with the cobalt oxide-based **CCG** nanocomposites, the Mn_3_O_4_–layered MnO_2_–N-doped rG-O nanocomposite is much more promising electrode material because of the low price and low toxicity of Mn elements. This result underscores the usefulness of the present synthetic strategy for exploring novel efficient electrode materials highly suitable for practical use.

## Discussion

In the present study, we are successful in developing a very efficient method to improve the electrode performance of graphene-based nanocomposite materials using the colloidal mixture of layered metal oxide and graphene nanosheet as a precursor. In fact, there are considerable numbers of reports about the cobalt oxide–graphene nanocomposite electrode materials for lithium secondary batteries^12^,^35^,^40^,^41^,^42^,^43^. In one instance, Wang *et al.* reports the large discharge capacity of 1200 mAh g^−1^ for the Co_3_O_4_–graphene film, which is greater than the data of other previous reports^12^,^40^. In comparison with these data, the present **CCG** nanocomposites show much larger discharge capacity of ~1550–1750 mAh g^−1^ with excellent cyclability and good rate characteristics, which outperforms all of the previously reported Co_3_O_4_-based electrode materials (Supplementary Information, Table S3). Similarly the electrode performance of the Mn_3_O_4_–layered MnO_2_–N-doped rG-O nanocomposite is superior to all the reported data of Mn_3_O_4_–graphene nanocomposite (Supplementary Information, Table S4). This result provides strong evidence for the unique merit of metal oxide nanosheets as an additive for graphene-based nanocomposite electrode materials. The observed dramatic enhancement of the electrode performance upon the incorporation of layered metal oxide nanosheet is attributable to the increase of surface area, the enhancement of the nanoscale mixing of components, the improvement of electrical transport properties, and the enhancement of morphological stability upon the incorporation of layered metal oxide nanosheets. The present study obviously verifies a beneficial and universal role of exfoliated metal oxide nanosheets in optimizing the electrode performance of graphene-based nanocomposite. As mentioned in the introduction section, the graphene-based nanocomposites boast versatile applications such as electrodes for secondary batteries, supercapacitors, fuel cells, and solar cells, photocatalysts, redox catalysts, nanobio materials, structural materials, and so on^4^,^5^,^6^,^7^,^8^,^11^,^15^,^40^,^41^,^42^,^43^,^44^,^45^,^46^. For most applications of these graphene-containing materials, the homogeneous blending between graphene nanosheets and hybridized functional materials, and the optimization of porous structure are commonly important in enhancing their functionalities. Taking into account the fact that the present synthetic strategy is readily applicable for many types of rG-O-based nanocomposites, the incorporation of exfoliated inorganic nanosheets can provide a powerful methodology to optimize the diverse functionalities of graphene-containing nanocomposites through the effective deterioration of the tight packing structure of graphene nanosheets. Our current research project is the use of the various colloidal mixtures of graphene and inorganic nanosheets for the exploration of novel graphene-based functional materials with various applicabilities for solar cells, photocatalyst, supercapacitors, and so on.

## Methods

### Materials preparation

The exfoliated layered CoO_2_ nanosheets was prepared by the proton exchange of LiCoO_2_ and intercalation of tetramethylammonium (TMA) ions into HCoO_2_^23^. The colloidal suspension of graphene oxide (G-O) was synthesized from graphite by a modified Hummers' method, in which the concentration of KMnO_4_ was reduced to 1/6 of conventional concentration^47^. The as-prepared G-O was dispersed in anhydrous ethanol with the concentration of 0.32 mg mL^−1^ by ultrasonication for 0.5 h. An aqueous suspension of exfoliated G-O (48 mL) was reacted with 2.4 mL 0.2 M Co(Ac)_2_ and 1 mL 30% NH_4_OH aqueous solution, 1 mL H_2_O, and layered CoO_2_ nanosheets (0–2 wt% to exfoliated G-O nanosheets). The mixture was stirred at 80 °C for 10 h. Then the mixture was transferred to an autoclave for hydrothermal reaction. The reaction condition was 3 h at 150 °C. In this step, G-O was reduced to N-doped rG-O. After the reaction, powdery precipitates were collected by centrifugation, washed with ethanol and distilled water, and then freeze-dried. After the completion of the reaction, only transparent supernatant solution remained. No observation of Tyndall phenomenon for the supernatant solution clearly demonstrated the absence of any precursor colloidal particles in this solution. Additionally, no formation of precipitate upon the addition of hydroxide ions confirmed the complete incorporation of Co^2+^, CoO_2_, and graphene reactants into the precipitated nanocomposite materials. On the basis of the present findings, the weight ratios of the components in these materials could be estimated from the starting ratios of the reactants. The weight ratio of Co_3_O_4_:CoO_2_:rG-O components was estimated to 3.7:0:1 for **CCG0**, 3.7:0.007:1 for **CCG5**, 3.7:0.015:1 for **CCG10**, and 3.7:0.03:1 for **CCG20**, respectively. Since the weight of G-O component was much more convenient and precise to calculate than its molar concentration, the weight ratios of CoO_2_/G-O were applied for controlling the compositions of the present nanocomposites like many other studies about the graphene-based nanocomposites.

### Materials characterization

The crystal structures of the as-prepared **CCG** nanocomposites and their electrochemically cycled derivatives were analyzed by powder XRD analysis (Rigaku D/Max-2000/PC, Cu Kα radiation, 298 K) analysis. The crystal morphology of the present samples was examined by FE-SEM (JEOL JSM-6700F) and HR-TEM/SAED (Jeol JEM-2100F, an accelerating voltage of 200 kV). The spatial elemental distribution of the present materials was probed with EDS–elemental mapping analysis. XANES spectroscopic experiment was carried out at Co K-edge at the beam line 10C at the Pohang Accelerator Laboratory (PAL) in Korea. The chemical bonding nature of nitrogen species was investigated with XPS analysis (Thermo VG, UK, Al Kα), in which a monochromated X-ray beams was used. All the XPS spectra were calibrated with a reference to the adventitious C 1s peak at 284.8 eV to rule out any possible spectral shift by the charging effect. To avoid the accumulation of charge during the measurement, all the samples were deposited on metallic copper foil. N_2_ adsorption–desorption isotherms were measured at 77 K using Micromeritics ASAP 2020 analyzer to determine the surface area. Before the measurements, the degassing of the samples was carried out at 150 °C for 3 h under vacuum. Micro-Raman spectra were obtained with a JY LabRam HR spectrometer using an excitation wavelength of 514.5 nm. The zeta potentials of the pure colloidal suspensions of G-O and layered CoO_2_ nanosheets, and their colloidal mixtures were measured with Malvern Zetasizer Nano ZS (Malvern, UK).

### Electrochemical measurement

The CV data were collected using an IVIUM analyzer with a scanning rate of 0.5 mV s^−1^ and a potential range of 0.01–3.0 V (*vs.* Li/Li^+^). The EIS data were collected in the frequency range of 0.01 Hz–100 KHz. Electrochemical measurements were carried out at room temperature using 2016 coin-type cell of 1 M LiPF_6_ in an equivolume mixture of ethylene carbonate/diethyl carbonate (EC/DEC = 50:50). The working electrodes were fabricated by mixing 80 wt% active material, 10 wt% Super P, and 10 wt% polyvinylidene fluoride (PVDF) dissolved in N-methyl-2-pyrrolidinone (NMP). The composite electrodes were prepared by coating the anode slurry onto a copper foil as a current collector and drying under vacuum at 110 °C for 12 h. The test cells were assembled in an argon-filled glove box. All the galvanostatic charge–discharge tests were performed with Maccor (Series 4000) multichannel galvanostat/potentiostat in the voltage range of 0.01–3.0 V (vs. Li/Li^+^) at current density of 100–3200 mA.

## Additional Information

**How to cite this article**: Jin, X. *et al.* An Effective Way to Optimize the Functionality of Graphene-Based Nanocomposite: Use of the Colloidal Mixture of Graphene and Inorganic Nanosheets. *Sci. Rep.* **5**, 11057; doi: 10.1038/srep11057 (2015).

## Supplementary Material

Supplementary Information
