# Supplementary material for: An Effective Way to Optimize the Functionality of Graphene-Based Nanocomposite: Use of the Colloidal Mixture of Graphene and Inorganic Nanosheets
Source: Sci Rep. 2015 Jun 8;5:11057. doi: 10.1038/srep11057 (PMC5377538; doi:10.1038/srep11057)
Supplement: Supplementary Information [file srep11057-s1.doc]

Supplementary Information

An Effective Way to Optimize the Functionality of Graphene-Based Nanocomposite: Use of the Colloidal Mixture of Graphene and Inorganic Nanosheets

Xiaoyan Jin^1^, Kanyaporn Adpakpang^1^, In Young Kim^1^, Seung Mi Oh^1^, Nam-Suk Lee^2^, & Seong-Ju Hwang^1^,*

^1^Department of Chemistry and Nanoscience, College of Natural Sciences, Ewha Womans University, Seoul 120-750, Korea

^2^National Institute for Nanomaterials Technology (NINT), Pohang University of Science and Technology (POSTECH), Pohang 790-784, Korea

* To whom all correspondences should be addressed

Tel) +82-2-3277-4370

Fax) +82-2-3277-3419

E-mail) hwangsju@ewha.ac.kr

**Supplementary Figure S1.** (A) Photoimages of (a) the colloidal suspension of G-O, (b) the colloidal suspension of layered CoO_2_ nanosheets, and the mixture colloidal suspensions of G-O and layered CoO_2_ nanosheets with the CoO_2_/G-O ratios of (c) 0.5wt%, (d) 1wt%, and (e) 2wt%, (f) the colloidal suspension of G-O after the NH_4_OH addition, (g) the colloidal suspension of layered CoO_2_ nanosheets after the NH_4_OH addition, and (h) the mixture colloidal suspension of G-O, layered CoO_2_ nanosheets and NH_4_OH after the NH_4_OH addition. (B) size distribution curve of exfoliated CoO_2_ nanosheets determined by the DLS analysis.


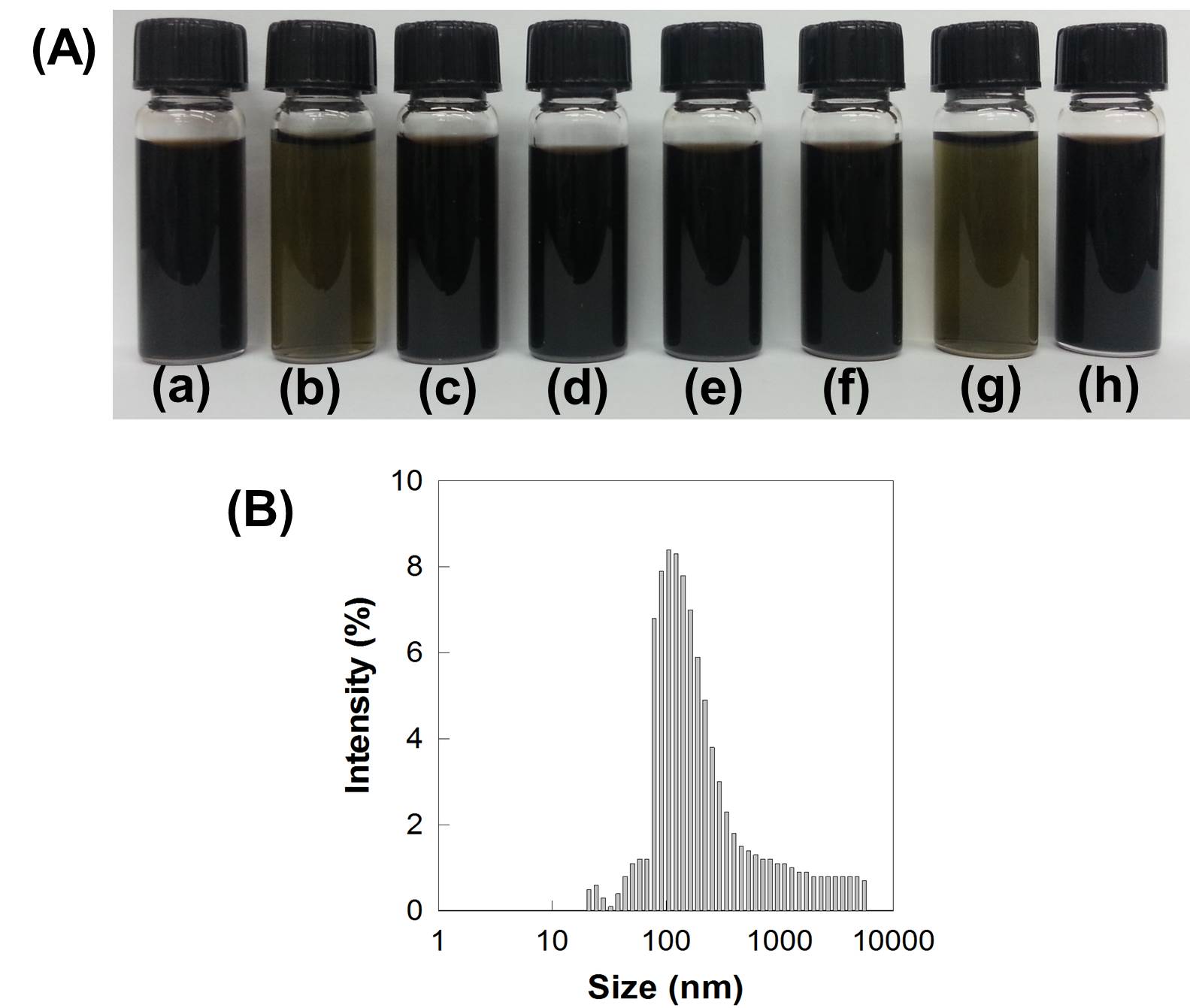


: As illustrated in **Fig. S1A**, both the exfoliated layered CoO_2_ and graphene oxide (G-O) nanosheets can form stable mixture colloidal suspensions with variable CoO_2_/G-O ratios of 0.5−2wt%, since two precursor nanosheets possess very similar surface charge and hydrophilicity each other. We examined the effect of NH_4_OH addition on the colloidal stability of G-O/CoO_2_ mixture as well as the pure colloidal suspensions of layered CoO_2_ and G-O nanosheets. Upon the addition of NH_4_OH, all the present colloidal suspensions remain unchanged without the formation of aggregated precipitates, clearly demonstrating the excellent colloidal stability of these suspensions. Additionally, the lateral dimension of the exfoliated CoO_2_ nanosheets is investigated with a standard dynamic light scattering (DLS) instrument. As presented in **Fig. S1B**, most of the exfoliated CoO_2_ nanosheets possess the lateral dimension of several hundreds of nanometers.

**Supplementary Table S1.** Zeta potential data of the G-O colloidal nanosheet, layered CoO_2_ colloidal nanosheet, and the mixture colloidal nanosheets of G-O and layered CoO_2_ with the CoO_2_/G-O ratios of 0.5wt%, 1wt%, and 2wt%.

| Material | Zeta potential (mV) |
| --- | --- |
| G-O colloid | -32.7 |
| CoO_2_ colloid | -46.8 |
| Mixture colloid with the CoO_2_/G-O ratio of 0.5wt% | -32.9 |
| Mixture colloid with the CoO_2_/G-O ratio of 1wt% | -33.5 |
| Mixture colloid with the CoO_2_/G-O ratio of 2wt% | -35.0 |

: As listed in **Table S1**, the mixture colloids of layered CoO_2_ and G-O nanosheets show fairly similar negative surface charge to those of the colloids of layered CoO_2_ and G-O nanosheets.

**Supplementary Figure S2.** (A) C 1s and (B) N 1s XPS spectra of (a) G-O, (b) **CCG0**, (c) **CCG5**, (d) **CCG10**, (e) **CCG20**, and (f) N-doped rG-O.


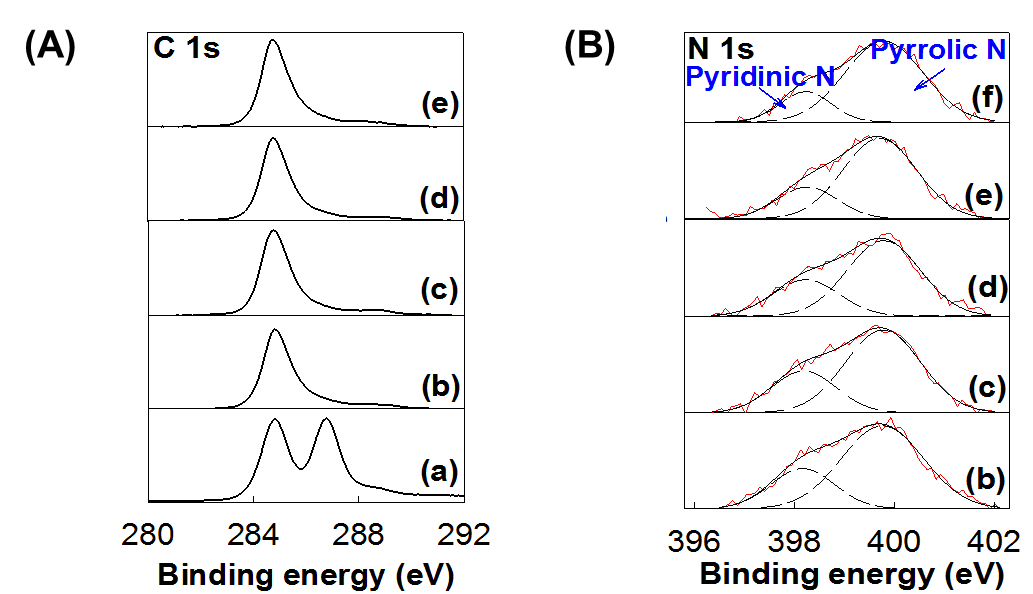


: The chemical bonding nature of graphene components in the present **CCG** nanocomposites is investigated with X-ray photoelectron spectroscopic (XPS) analysis. As shown in **Fig. S2A**, the C 1s spectra of the reference G-O obviously exhibit two strong peaks at ~285 and ~287 eV, which correspond to sp^2^ carbon of the basal plane of graphene and the oxygenated carbon species, respectively. Conversely only the lower binding energy peak is discernible at ~285 eV for all the present **CCG** nanocomposites, confirming the efficient reduction of G-O to reduced graphene oxide (rG-O) during the hydrothermal synthesis. In addition, the N doping of the graphene component in the **CCG** nanocomposites is clearly verified with the observation of distinct N 1s XPS signal, see Fig. S2B. Regardless of the CoO_2_ content incorporated, most of nitrogen species in the **CCG** nanocomposites exist as pyrrolic N with smaller amount of pyridinic N (**Table S2**), as reported previously^1^.

**Supplementary Table S2.** Relative concentrations of nitrogen species in the **CCG** nanocomposites determined from N 1s XPS results.

| Material | Pyridinic N | Pyrrolic N |
| --- | --- | --- |
| N-doped rG-O nanosheet | 25.7% | 74.3% |
| CCG0 | 26.0% | 74.0% |
| CCG5 | 30.0% | 70.0% |
| CCG10 | 29.5% | 70.5% |
| CCG20 | 27.8% | 72.8% |

: The chemical bonding nature of doped nitrogen in the present **CCG** nanocomposites is examined with peak deconvolution analysis for the N 1s XPS data. As listed in **Table S2**, regardless of layered CoO_2_ content incorporated, most of nitrogen species in the **CCG** nanocomposites exist as pyrrolic N with smaller amount of pyridinic N.

**Supplementary Figure S3.** EDS−elemental mapping data and the corresponding FE-SEM images of the nanocomposites of (a) **CCG0**, (b) **CCG5**, (c) **CCG10**, and (d) **CCG20**.


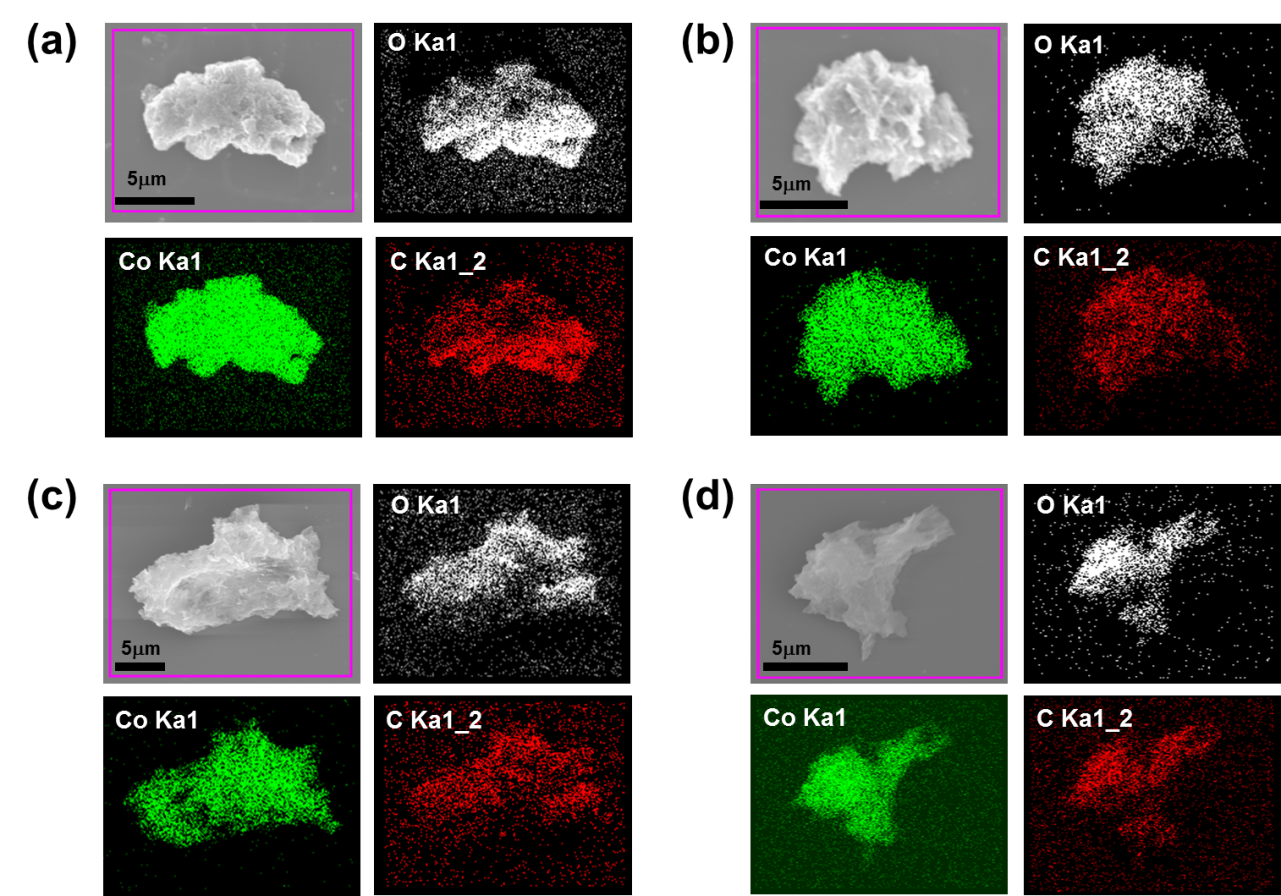


: As illustrated in **Fig. S3,** the nanoscale hybridization between cobalt oxides and reduced graphene oxide (rG-O) nanosheets in the present Co_3_O_4_−layered CoO_2_−N-doped rG-O (**CCG**) nanocomposites is confirmed by energy-dispersive X-ray spectroscopy (EDS)−elemental mapping analysis with field emission-scanning electron microscopic (FE-SEM) images, showing the uniform distribution of cobalt, oxygen, and carbon elments in entire parts of the nanocomposite materials.

**Supplementary Figure S4.** (Left) Powder XRD pattern and (right) FE-SEM image of the rG-O-free Co_3_O_4_−layered CoO_2_ nanocomposite.


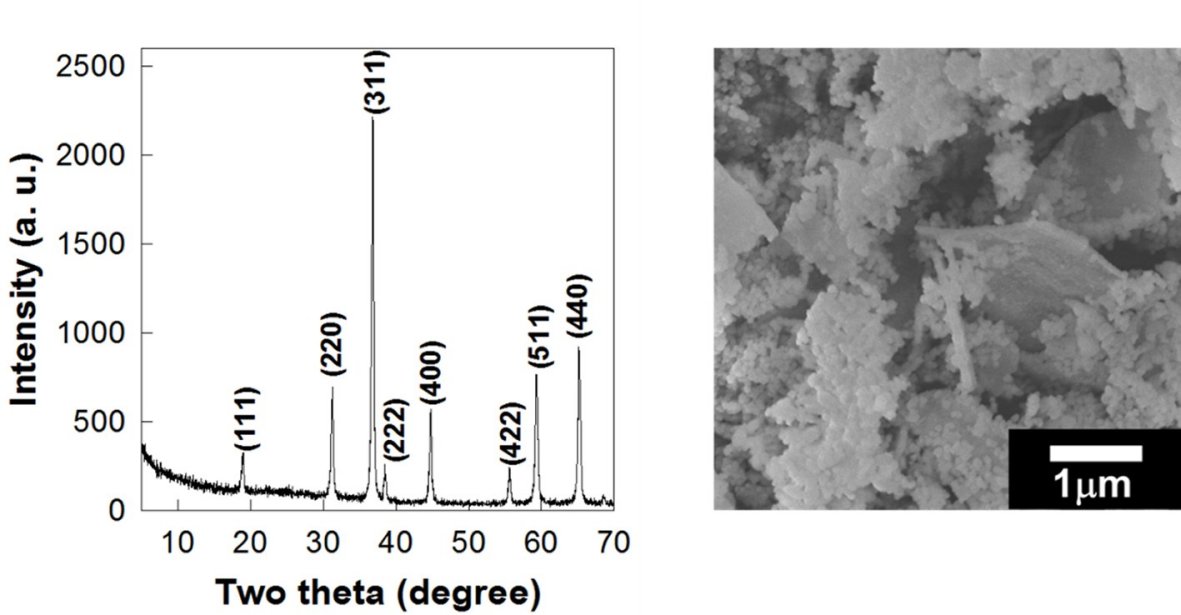


: For comparison, the rG-O-free Co_3_O_4_−layered CoO_2_ nanocomposite was also synthesized by the same synthetic method as that for the present **CCG** nanocomposites except for the absence of the G-O precursor and ammonia solution. As plotted in the left panel of **Fig. S4**, the obtained Co_3_O_4_−layered CoO_2_ nanocomposite shows typical X-ray diffraction (XRD) peaks of spinel-structured Co_3_O_4_ phase, as observed for the present **CCG** nanocomposites. This finding confirms the formation of mixed valent cobalt (II,III) oxide phase during the hydrothermal reaction. As presented in FE-SEM image of the right panel of **Fig. S4**, this nanocomposite displays porous morphology formed by the house-of-cards-type stacking structure. The spherical Co_3_O_4_ nanoparticles are anchored on the surface of layered CoO_2_ nanosheets. This presnet result clearly demonstrates that the layered CoO_2_ nanosheet can play a role of support for the anchoring of Co_3_O_4_ nanoparticles.

**Supplementary Figure S5.** Co 2p XPS spectra of (a) Co_3_O_4_, (b) **CCG0,** (c) **CCG5,** (d) **CCG10,** and (e) **CCG20.**


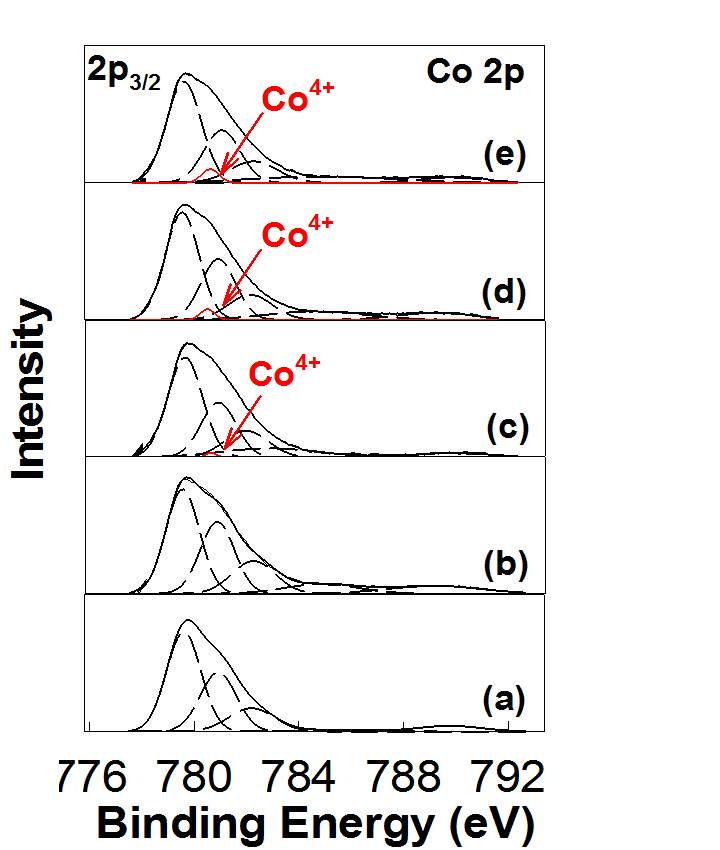


: **Fig. S5** represents the results of peak convolution analysis for the Co 2p_3/2_ X-ray photoelectron spectra (XPS) of the **CCG** nanocomposites and the reference Co_3_O_4_. According to the previous literatures^2,3^, the peaks at 779.8, 780.7, and 781.2 eV can be assigned as Co^3+^, Co^4+^, and Co^2+^ ions, respectively. The tetrahedral local symmetry of Co^2+^ ion in Co_3_O_4_ leads to the higher binding energy compared with the Co^3+^ and Co^4+^ ions with octahedral symmetry^2,3^. The additional peaks beyond 782.5 correspond to the shake-up satellite peaks of Co^2+^ ion. As can be seen clearly from **Fig. S5**, the tetravalent Co^4+^ ions are obviously identified and the concentration of Co^4+^ ion becomes greater with increasing the concentration of CoO_2_ nanosheet incorporated. This result provides further evidence for the presence of CoO_2_ nanosheets in the present **CCG** composite.

**Supplementary Table S3**. Physical properties and electrochemical Li cycling data of Co_3_O_4_-based electrode materials in the previous reports.

| Material & morphology | Current density (mAg^−1^) | Discharge capacity after the nth cycle (mAhg^−1^) | Ref |
| --- | --- | --- | --- |
| Co_3_O_4_ nanocage | 50 | 970 (n=30) | 4 |
| mesoporous frameworks Co_3_O_4_ nanoparticles | 1C | 814 (n=50) | 5 |
| Co_3_O_4_ hollow microsphere | 50 | 1615 (n=30) | 6 |
| Co_3_O_4_ nanobelt | 100 | 980 (n=60) | 7 |
| hierarchical structured Co_3_O_4_/C | 100 | 1035 (n=50) | 8 |
| Co_3_O_4_/graphene | 50 | 935 (n=30) | 9 |
| Co_3_O_4_/C hollow nanosphere | 0.25C | 1150 (n=30) | 10 |
| Co_3_O_4_/reduced graphene oxide | 40 | 860 (n=120) | 11 |
| Co_3_O_4_/graphene composite | 74 | 760 (n=20) | 12 |
| Co_3_O_4_/N−graphene | 400 | 800 (n=50) | 13 |
| Co_3_O_4_−graphene sheet-on-sheet | 0.1C | 1065 (n=30) | 14 |
| Co_3_O_4_/graphene film | 200 | 1200 (n=100) | 15 |
| peapod−like Co@carbon to Co_3_O_4_ @carbon | 1C | 1050 (n=50) | 16 |
| Co_3_O_4_ and CoO@C | 100 | 1240 (n=70) | 17 |
| Co_3_O_4_ nanotubes on CNT | 50 | 1200 (n=70) | 18 |
| Co_3_O_4_ /rGO film | 50 | 1108 (n=50) | 19 |
| Co_3_O_4_−C core-shell nanowire | 0.5C | 989 (n=50) | 20 |
| Co_3_O_4_@graphene membrane | 500 | 600 (n=500) | 21 |
| Co_3_O_4_/MWCNT | 0.2C | 940 (n=70) | 22 |
| Co_3_O_4_/Fe_2_O_3_ nanowire | 200 | 1005 (n=50) | 23 |
| CuO/Co_3_O_4_ composite | 60 | 873 (n=40) | 24 |

**Supplementary Figure S6.** Potential profiles at current density of 200 mAhg^−1^ for the nanocomposites of (a) **CCG0**, (b) **CCG5**, (c) **CCG10**, (d) **CCG20**, and (e) layered CoO_2_ nanosheets.


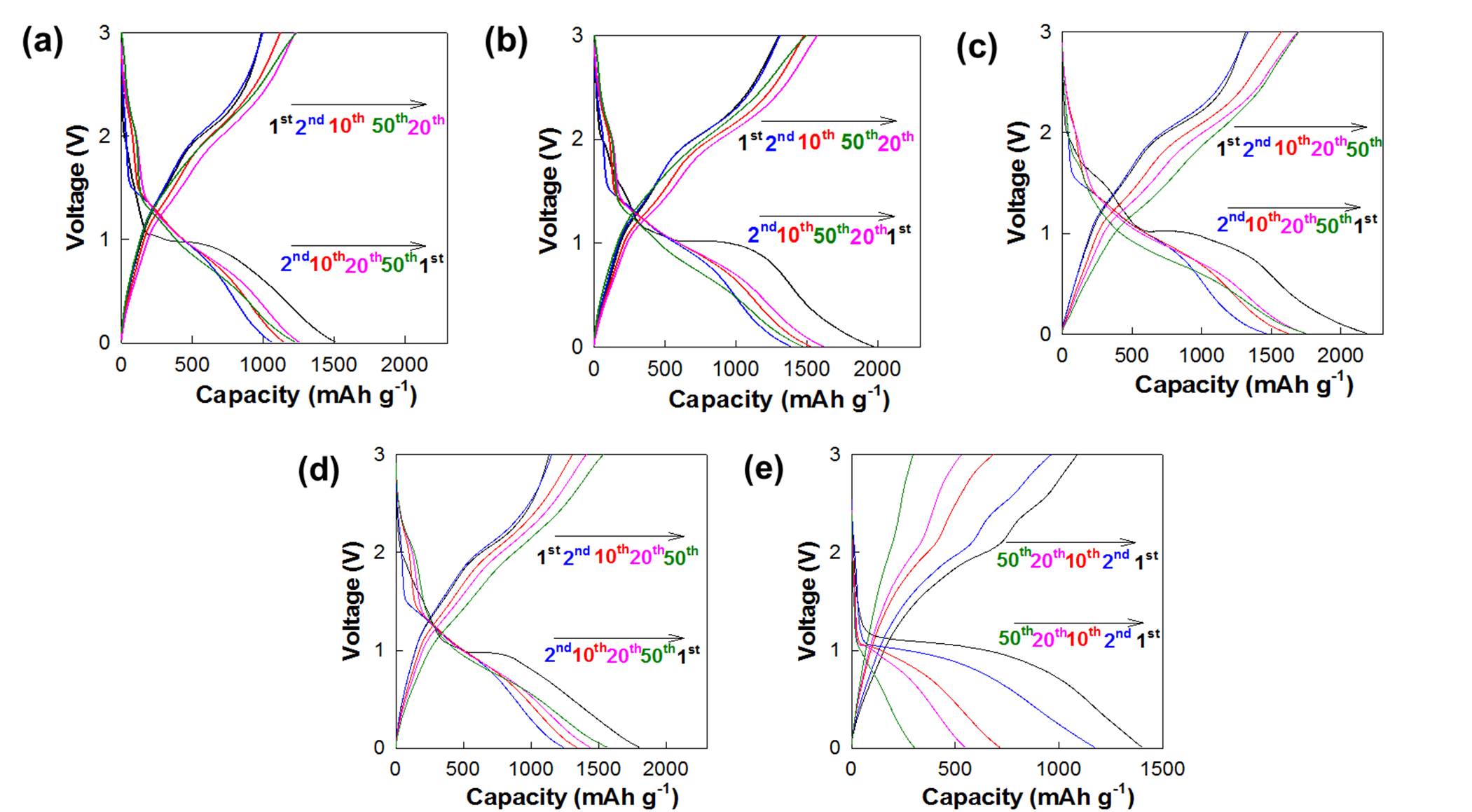


: As plotted in **Fig. S6**, all the present **CCG** nanocomposites exhibit nearly identical potential profiles to that of Co_3_O_4_ phase. This observation clearly demonstrates that the electrochemical activity of the **CCG** nanocomposites mainly originates from the Co_3_O_4_ component.

**Supplementary Figure S7.** (a) Powder XRD pattern, (b) FE-SEM image, (c) discharge−charge capacity plot, and (d) rate-dependant capacity plot of the Mn_3_O_4_−MnO_2_−N-doped rG-O nanocomposite measured at current density of 200 mA g^−1^.


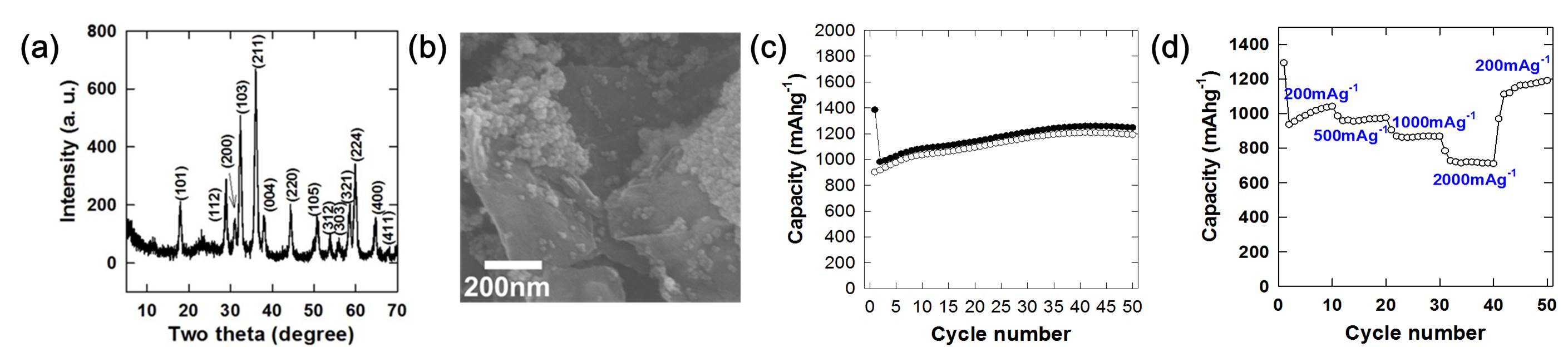


: To probe the universal usefulness of exfoliated metal oxide nanosheet as an additive in improving the electrode performance of other metal oxide−graphene nanocomposites, multicomponent Mn_3_O_4_−layered MnO_2_−N-doped rG-O nanocomposite was synthesized by the same method as that for the present **CCG** nanocomposite. As a precursor, the exfoliated layered MnO_2_ nanosheet was prepared by the proton exchange of K_0.45_MnO_2_ and the following intercalation of tetrabutylammonium (TBA) ions into the protonated derivative^25^. Another precursor, the colloidal suspension of G-O, was synthesized from graphite by a modified Hummers' method. The as-prepared G-O was dispersed in anhydrous ethanol with the concentration of 0.32 mg mL^−1^ by ultrasonication for 0.5 h. An aqueous suspension of exfoliated G-O (48 mL) was reacted with 2.4 mL 0.2 M Mn(Ac)_2_, 1 mL 30% NH_4_OH aqueous solution, 1 mL H_2_O, and layered MnO_2_ nanosheets (2.5wt% to exfoliated G-O nanosheets). As plotted in the XRD pattern of **Fig. S7a**, the obtained Mn_3_O_4_−layered MnO_2_−N-doped rG-O nanocomposite shows typical Bragg reflections of spinel-structured Mn_3_O_4_ phase, confirming the formation of mixed valent manganese (II,III) oxide phase during the hydrothermal reaction. The present material shows porous morphology, which is formed by the house-of-cards-type stacking structure. The present nanocomposite displays fairly promising electrode performance with the discharge capacity of 1250 mAh g^−1^ at the 50th cycle, see the **Fig. S7c**. The current density-dependent discharge capacities of the Mn_3_O_4_−layered MnO_2_−N-doped rG-O nanocomposite are also measured. As plotted in **Fig. S7d,** this nanocomposite material can still deliver a capacity of ~700 mAh g^−1^ even at high current density of 2000 mAg^−1^, underscoring its excellent electrode performance. Although the future optimization of synthesis condition can induce a further improvement of the electrode performance of the present Mn_3_O_4_−layered MnO_2_−N-doped rG-O nanocomposite, the electrode performance presented here is much superioir to previously reported data of Mn_3_O_4_−graphene nanocomposites, as listed in **Table S4**.

**Supplementary Table S4**. Physical properties and electrochemical Li cycling data of Mn_3_O_4_-based electrode materials in the previous reports.

| Material & morphology | Current density (mAg^−1^) | Discharge capacity after the nth cycle (mAhg^−1^) | Ref |
| --- | --- | --- | --- |
| Mn_3_O_4_−reduced graphene oxide | 40 | 900 (n=5) | 26 |
| Mn_3_O_4_−graphene nanocomposite | 40 | 900 (n=50) | 27 |
| Mn_3_O_4_/graphene | 60 | 645 (n=40) | 28 |
| Mn_3_O_4_/graphene platelet/LiCMC composite | 75 | 720 (n=100) | 29 |
| Mn_3_O_4_/graphite nanosheet | 200 | 437 (n=50) | 30 |
| carbon layer coated Mn_3_O_4_ nanorod | 40 | 473 (n=50) | 31 |
| spongelike nanosized Mn_3_O_4_ | 0.25C | 800 (n=40) | 32 |
| Mn_3_O_4_−N-doepd graphene | 200 | 703 (n=40) | 33 |
| 3D Mn_3_O_4_/ordered mesoporous carbon | 100 | 802 (n=50) | 34 |
| Mn_3_O_4_@C | 40 | 550 (n=50) | 35 |

References for supplementary data

1. Liang, Y. *et al*. Co_3_O_4_ nanocrystals on graphene as a synergistic catalyst for oxygen reduction reaction. *Nat. Mater.* **10**, 780−786 (2011).
2. Chuang, T. J., Brundle, C. R. & Rice, D. W. Interpretation of the x-ray photoemission spectra of cobalt oxides and cobalt oxide surfaces. *Surf. Sci.* **59**, 413−429 (1976).
3. Dupin, J. C., Gonbeau, D., Benqlilou-Moudden, H., Vinatier, Ph. & Levasseur, A. XPS analysis of new lithium cobalt oxide thin-films before and after lithium deintercalation. *Thin Solids Films* **384**, 23−32 (2001).
4. Yan, N. *et al.* Nanocages for high-performance anode material in lithium-ion batteries. *J. Phys. Chem. C* **116**, 7227−7235 (2012).
5. Fan, S. *et al.* Non-aqueous synthesis of crystalline Co_3_O_4_ nanoparticles for lithium-ion batteries. *Mater. Lett.* **91**, 291−293 (2013).
6. Wang, J. *et al.* Accurate control of multishelled Co_3_O_4_ hollow microspheres as high-performance anode materials in lithium-ion batteries. *Angew. Chem. Int. Ed.* **52**, 6417−6420 (2013).
7. Huang, H. *et al.* Nanocrystal-constructed mesoporous single-crystalline Co_3_O_4_ nanobelts with superior rate capability for advanced lithium-ion batteries. *ACS Appl. Mater. Interf.* **4**, 5974−5980 (2012).
8. Sun, J., Liu, H., Chen, X., Evans, D. G. & Yang, W. An oil droplet template method for the synthesis of hierarchical structured Co_3_O_4_/C anodes for Li-ion batteries. *Nanoscale* **5**, 7564−7571 (2013).
9. Wu, Z. -S. *et al.* Graphene anchored with Co_3_O_4_ nanoparticles as anode of lithium ion batteries with enhanced reversible capacity and cyclic performance. *ACS Nano* **4**, 3187−3194 (2010).
10. Liu, J. *et al.* Solvothermal synthesis of uniform Co_3_O_4_/C hollow quasi-nanospheres for enhanced lithium ion intercalation applications. *Eur. J. Inor. Chem.* **24**, 3825−3829 (2012).
11. Pan, L., Zhao, H., Shen, W., Dong, X. & Xu, J. Surfactant-assisted synthesis of a Co_3_O_4_/reduced graphene oxide composite as a superior anode material for Li-ion batteries. *J. Mater. Chem. A* **1**, 7159**−**7166 (2013).
12. Yang, S. *et al.* Fabrication of cobalt and cobalt oxide/graphene composites: towards high-performane anode materials for lithium ion batteries. *ChemSusChem* **3**, 236−239 (2010).
13. Li, D. *et al.* Enhanced rate performance of cobalt oxide/nitrogen doped graphene composite for lithium ion batteries. *RSC Adv.* **3,** 5003−5008 (2013).
14. Chen, S. Q. & Wang, Y. Microwave-assisted synthesis of a Co_3_O_4_−graphene sheet-on-sheet nanocomposite as a superior anode material for Li-ion batteries. *J. Mater. Chem.*  **20,** 9735−9739 (2010).
15. Wang, R. *et al.* Free-standing and binder-free lithium-ion electrodes based on robust layered assembly of graphene and Co_3_O_4_ nanosheets. *Nanoscale* **5**, 6960−6967 (2013).
16. Wang, Y. *et al.* Designed functional systems from peapod-like Co@carbon to Co_3_O_4_@carbon nanocomposites. *ACS Nano* **4**, 4753−4761 (2010).
17. Xiong, S., Chen, J. S., Lou, X. W. & Zeng, H. C. Mesoporous Co_3_O_4_ and CoO@C topotactically transformed from chrysanthemum-like Co(CO_3_)_0.5_(OH)·0.11H_2_O and their lithium-storage properties. *Adv. Funct. Mater.* **22**, 861−871 (2012).
18. Du, N. *et al.* Porous Co_3_O_4_ nanotubes derived from Co_4_(CO)_12_ clusters on carbon nanotube templates: A highly efficient material for Li-battery applications. *Adv. Mater.* **19**, 4505−4509 (2007).
19. Choi, B. G. *et al.* 3D heterostructured architectures of Co_3_O_4_ nanoparticles deposited on porous graphene surfaces for high performance of lithium ion batteries. *Nanoscale* **4**, 5924−5930 (2012).
20. Chen, J. *et al.* Co_3_O_4_−C core-shell nanowire array as an advanced anode material for lithium ion batteries. *J. Mater. Chem.* **22**, 15056−15061 (2012).
21. Li, L. *et al.* Co_3_O_4_  mesoporous nanostructures@graphene membrane as an integrated anode for long-life lithium-ion batteries. *J. Power Sources* **255**, 52−58 (2014).
22. Fang, Z. *et al.* Facile scalable synthesis of Co_3_O_4_/carbon nanotube hybrids as superior anode materials for lithium-ion batteries. *Mater. Res. Bull.* **48**, 4419−4423 (2013).
23. Xiong, Q. Q. *et al.* Hierarchical Fe_2_O_3_@Co_3_O_4_  nanowire array anode for high-performance lithium-ion batteries. *J. Power Sources* **240**, 344−350 (2013).
24. Reddy, M. V., Yu, C., Jiahuan, F., Loh, K. P. & Chowdari, B. V. R. Molten salt synthesis and energy storage studies on CuCo_2_O_4_ and CuO·Co_3_O_4_. *RSC Adv.* **2**, 9619−9625 (2012).
25. Omomo, Y., Sasaki, T., Wang, L. & Watanabe, M. Redoxable nanosheet crystallites of MnO_2_ derived via delamination of a layered manganese oxide. *J. Am. Chem. Soc.* **125**, 3568−3575 (2003).
26. Wang, H. *et al.* Mn_3_O_4_−graphene hybrid as a high-capacity anode material for lithium ion batteries. *J. Am. Chem. Soc.* **132**, 13978−13980 (2010).
27. Li, L., Guo, Z., Du, A. & Liu, H. Rapid microwave-assisted synthesis of Mn_3_O_4_−graphene nanocomposite and its lithium storage properties. *J. Mater. Chem.* **22**, 3600−3605 (2012).
28. Nam, I., Kim, N. D., Kim, G. -P., Park, J. & Yi, J. One step preparation of Mn_3_O_4_/graphene composites for use as an anode in Li ion batteries. *J. Power Sources* **244**, 56−62 (2013).
29. Lavoie, N., Malenfant, P. R. L., Courtel, F. M., Abu-Lebdeh, Y. & Davidson, I. J. High gravimetric capacity and long cycle life in Mn_3_O_4_/graphene platelet/LiCMC composite lithium-ion battery anodes. *J. Power Sources* **213**, 249−254 (2012).
30. Liu, S. -Y. *et al.* Nanocrystal manganese oxide (Mn_3_O_4_, MnO) anchored on graphite nanosheet with improved electrochemical Li-storage properties. *Electrochim. Acta* **66**, 271−278 (2012).
31. Wang, C., Yin, L., Xiang, D. & Qi, Y. Uniform carbon layer coated Mn_3_O_4_ nanorod anodes with improved reversible capacity and cyclic stability for lithium ion batteries. *ACS Appl. Mater. Interf.* **4**, 1636−1642 (2012).
32. Gao, J., Lowe, M. A. & Abruña, H. D. Spongelike nanosized Mn_3_O_4_ as a high-capacity anode material for rechargeable lithium batteries. *Chem. Mater.* **23**, 3223−3227 (2011).
33. Park, S. -K *et al. In situ* hydrothermal synthesis of Mn_3_O_4_ nanoparticles on nitrogen-doped graphene as high-performance anode materials for lithium ion batteries. *Electrochim. Acta* **120**, 452−459 (2014).
34. Li, Z. *et al.* Three-dimensional nanohybrids of Mn_3_O_4_/ordered mesoporous carbons for high performance anode materials for lithium-ion batteries*. J. Mater. Chem.* **22**, 16640−16648 (2012).
35. Wang, C., Yin, L., Xiang, D. & Qi, Y. Uniform carbon layer coated Mn_3_O_4_ nanorod anodes with improved reversible capacity and cyclic stability fo lithium ion batteries. *ACS Appl. Mater. Interf.* **4**, 1636−3605 (2012).
